# Gait Device Treatment Using Telehealth for Individuals With Stroke During the COVID-19 Pandemic: Nonrandomized Pilot Feasibility Study

**DOI:** 10.2196/43008

**Published:** 2023-05-19

**Authors:** Brianne Darcy, Lauren Rashford, Stephen Tyler Shultz, Nancey T Tsai, David Huizenga, Kyle B Reed, Stacy J M Bamberg

**Affiliations:** 1 Moterum Technologies Salt Lake City, UT United States; 2 Department of Physical Therapy Wingate University Wingate, NC United States; 3 Department of Mechanical Engineering University of South Florida Tampa, FL United States

**Keywords:** gait device, telerehabilitation, iStride, stroke rehabilitation, walking speed, gait, gait treatment, telehealth, COVID-19

## Abstract

**Background:**

During the COVID-19 pandemic, rehabilitation providers and consumers adopted telehealth practices at unprecedented rates. Multiple prepandemic studies demonstrate the feasibility and comparable efficacy between in-clinic and remote treatment for certain impairments caused by stroke, such as upper extremity weakness and impaired motor function. However, less guidance has been available regarding gait assessment and treatment. Despite this limitation, safe and effective gait treatment is fundamental to optimizing health and well-being after stroke and should be considered a treatment priority, including during the COVID-19 pandemic.

**Objective:**

This study explores the feasibility of using telehealth to deliver gait treatment using a wearable gait device, the iStride device, to stroke survivors during the 2020 pandemic. The gait device is used to treat hemiparetic gait impairments caused by stroke. The device alters the user’s gait mechanics and creates a subtle destabilization of the nonparetic limb; therefore, supervision is required during its usage. Before the pandemic, treatment with the gait device had been provided in person to appropriate candidates using a combination of physical therapists and trained personnel. However, upon the emergence of the COVID-19 pandemic, in-person treatment was halted in adherence to pandemic guidelines. This study investigates the feasibility of 2 remote delivery treatment models with the gait device for stroke survivors.

**Methods:**

Participants were recruited during the first half of 2020 after the onset of the pandemic and included 5 individuals with chronic stroke (mean age 72 years; 84 months post stroke). Four participants were previous gait device users who transitioned to the telehealth delivery model to continue their gait treatment remotely. The fifth participant performed all study-related activities, from recruitment through follow-up, remotely. The protocol included virtual training for the at-home care partner, followed by 3 months of remote treatment with the gait device. Participants were instructed to wear gait sensors during all treatment activities. To assess feasibility, we monitored the safety of the remote treatment, compliance with protocol activities, acceptability of the telehealth treatment delivery, and preliminary efficacy of the gait treatment. Functional improvement was measured using the 10-Meter Walk Test, the Timed Up and Go Test, and the 6-Minute Walk Test, and quality of life was assessed using the Stroke-Specific Quality of Life Scale.

**Results:**

No serious adverse events occurred, and participants rated high acceptance of the telehealth delivery. Protocol compliance averaged 95% of treatment sessions, 100% of assessments, and 85% of sensor usage during treatment. After 3 months of treatment, the average improvement in each functional outcome exceeded the minimal clinically important difference or minimal detectable change value.

**Conclusions:**

Remote treatment delivery with the gait device appeared feasible with care partner support. Gait treatment using telehealth may be useful to offset negative immobility impacts for those requiring or preferring remote care during the pandemic or otherwise.

**Trial Registration:**

ClinicalTrials.gov NCT04434313; https://clinicaltrials.gov/ct2/show/NCT04434313

## Introduction

In the early months of 2020, rehabilitation services, like other facets of health care, were profoundly altered by the onset of the COVID-19 pandemic [1]. In an effort to mitigate disease spread to both patients and providers, telehealth technologies were implemented to facilitate remote access to health care, including rehabilitation [2,3], which was recognized as an “essential service” by the World Health Organization [4]. Among the populations specifically encouraged to pursue remote rehabilitation were those that were deemed vulnerable to severe illness during the pandemic. This group included individuals with stroke [5], who were found to have a 2.5-times increased risk of severe illness following COVID-19 infection [6].

Multiple prepandemic studies have indicated substantial promise in the realm of telerehabilitation for persons with stroke [7]. For example, a study published in JAMA Neurology [8] with 124 individuals experiencing arm motor deficits from a stroke showed that treatment using a home-based telerehabilitation system delivered comparable efficacy to treatment provided in a traditional clinic setting. Similarly, a study reviewing the usage of telerehabilitation for individuals with poststroke aphasia concluded that aphasia treatment delivered remotely appeared as effective as treatment delivered face-to-face [9]. Moreover, a systematic review by Tchero et al [10] concluded that telerehabilitation relayed equivalent outcomes for those with stroke in specific areas, including health-related quality of life, motor function, caregiver strain, and depression. While these and other promising studies suggest that comparable improvements can be achieved by treatments delivered in-person and remotely for certain impairments caused by stroke, less guidance has been available in the realm of gait treatment, perhaps due to the perceived complexities of navigating safety, mobility support, and gait pattern monitoring [11] remotely. However, while survivors of stroke commonly experience a variety of challenges, approximately 80% experience walking dysfunction [12], and only 25% return to a level of community participation equivalent to individuals without stroke [13]. To promote mobility independence, reduce the risk of falls, and enhance quality of life, access to safe and effective gait treatment is essential, including during the COVID-19 pandemic.

The iStride gait device [14] (Moterum Technologies, Inc) was designed to treat hemiparetic gait impairments resulting from stroke. A prior published study of 21 individuals with chronic stroke showed that 71% of participants improved their gait speed by a clinically meaningful amount, and 80% of participants reduced their risk for falls on at least one fall prediction outcome measure after 12 sessions of home-based treatment with the gait device [15]. The device alters the user’s gait mechanics and creates a subtle destabilization of the nonparetic limb; therefore, supervision is required during gait device usage. Published manuscripts describe the device’s mechanism in greater detail [16-18].

Before the COVID-19 pandemic, treatment with the gait device had been provided in person to appropriate candidates (within previous clinical trials and as rehabilitation treatment) using a combination of physical therapists and trained personnel. However, upon the development of the COVID-19 pandemic, in-person treatment was halted in adherence to Centers for Disease Control and Prevention (CDC) guidelines [19]. Therefore, the objective of this study was to investigate the feasibility of providing this intervention during the onset of the pandemic remotely using 2 different telehealth delivery models—one method involving a transition to the telehealth model after a period of in-person treatment, and a second method involving performing all activities related to the gait device treatment remotely. This study reports the processes and feasibility-related findings of approximately 3 months of remote gait device treatment for 5 individuals with chronic stroke. In addition to the insights gained from our feasibility findings, this methodological depiction may offer relevance to clinicians or researchers navigating the complexities of remote gait analysis and treatment.

## Methods

### Ethics Approval

Ethical approval for the study was granted by the Wingate University Research Review Board (reference number 04172020). The study was registered on ClinicalTrials.gov (NCT04434313). Although this is not a randomized pilot study, for thoroughness, the reporting of this study follows the extension of the CONSORT (Consolidated Standards of Reporting Trials) 2010 checklist, specifically pertaining to pilot trials [20]. Checklist items specific to randomization were excluded.

### Study Design

This study used a nonrandomized design to investigate the feasibility of delivering treatment with the gait device using telehealth. Two unique methodological processes were used across 5 participants and will be discussed in terms of the specific methodological processes for participants A-D (prior users of the gait device before the clinical trial’s inception) and the methodological processes for participant E (no previous experience with the gait device before consenting to the clinical trial). All aspects of the study were conducted in each participant’s home environment, with study staff interacting remotely with the participants using real-time video conferencing.

### Participants

#### Eligibility Overview

The eligibility criteria for treatment with the gait device in persons with chronic stroke have been specified below.

#### Inclusion Criteria

The inclusion criteria are as follows:

1. Age 21-80 years
2. One or more cerebral strokes (all on the same side)
3. Stroke occurred at least 3 months previously
4. Gait asymmetry but can walk independently with or without a cane (Modified Rankin Score 3 or less)
5. No evidence of severe cognitive impairment that would interfere with understanding instructions
6. No evidence of 1-sided neglect affecting ambulation
7. Adequate walking space within the home
8. Weight does not exceed 275 pounds

#### Exclusion Criteria

The exclusion criteria are as follows:

1. Uncontrolled seizures
2. Pregnancy
3. Metal implants (stents, clips, or pacemakers)
4. Chronic obstructive pulmonary disease
5. Uncontrolled blood pressure
6. Myocardial infarction within the last 180 days
7. Head injury within the last 180 days
8. A history of a neurologic disorder other than stroke

For the telehealth delivery models investigated in this clinical trial, we additionally required each participant to have support from a care partner (ie, caregiver) during all treatment sessions. This partner was to be an adult over the age of 18 years without physical or cognitive limitations, which would preclude them from being able to assist as needed.

#### Participants A-D: Hybrid Model

Participants A-D began rehabilitation treatment with the gait device in February 2020, several months before the inception of this trial. Their course of treatment at that time included a physical therapist-led assessment of device appropriateness, baseline in-person functional mobility testing, device training, and between 6 and 14 gait device sessions conducted in the individual’s home environment with trained personnel physically present. Upon the worsening of the COVID-19 pandemic, this clinical trial was approved by the Wingate University Research Review Board to comply with COVID-19 pandemic guidelines. Before commencing telehealth treatment, all participants provided written consent for study participation. Additionally, all individuals were refunded the lease price of their gait equipment (including the gait device and sensors) due to the investigative nature of the study.

#### Participant E: Fully Remote Model

Participant E underwent all processes using remote methods. A discussion of gait device treatment eligibility was initiated by a study team member via phone call. To further verify eligibility, video teleconferencing was used to confirm the safety of the home environment, including unobstructed walkways and stable flooring. Finally, a video gait review was used to assess baseline walking patterns in accordance with gait device eligibility criteria. A 3-person clinical trial physical therapist panel reviewed all participant details, including the video of the individual’s gait. Pending agreement by all 3 therapists on the appropriateness of gait device intervention using telehealth, the participant consented to study participation.

### Telehealth Protocol

#### Overview

**Figure 1:**
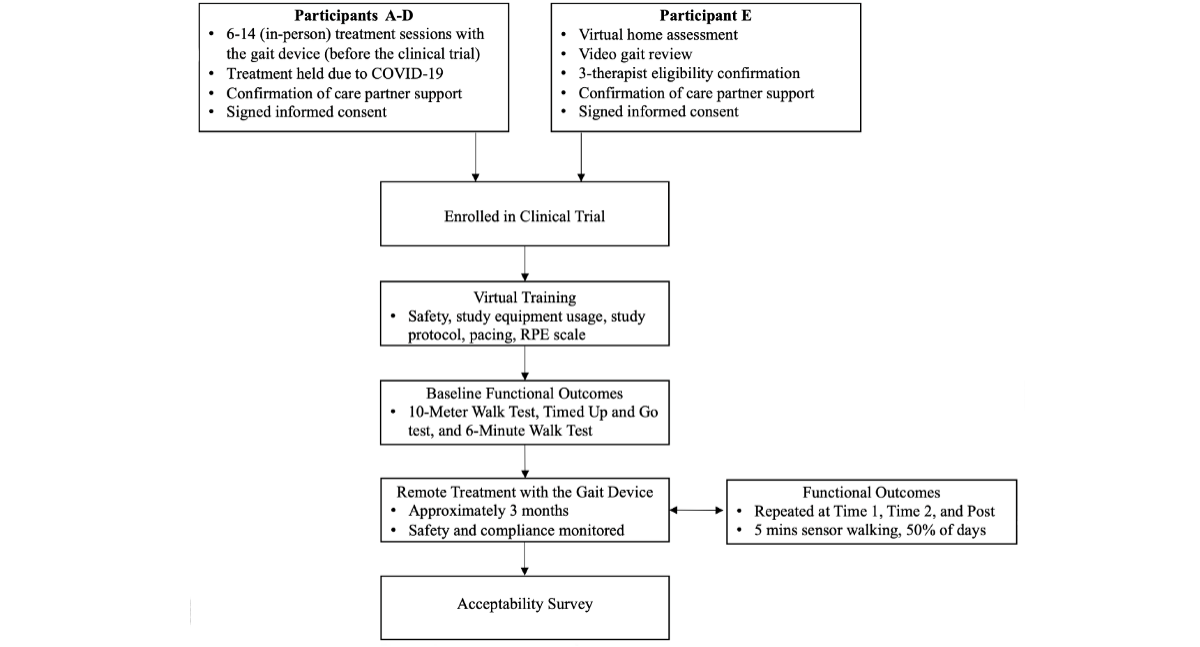
The sequence of activities for the clinical trial participants. Key activities for participants A-E are listed within each phase.

Study protocol oversight was performed by licensed physical therapists in accordance with physical therapy telehealth regulations. After consenting to participation, each participant and their care partner underwent a virtual training session followed by approximately 3 months of remote gait device treatment. Virtually conducted mobility assessments occurred at baseline, after 1 month of treatment, approximately halfway through the treatment, and after the conclusion of treatment. A telerehabilitation platform, the Moterum Digital Platform, was used to schedule and track the participant’s engagement with the protocol activities throughout the clinical trial. Further details of each protocol activity are discussed in greater detail below. All treatment was provided virtually throughout the clinical trial period, between July and December 2020. Figure 1 depicts the sequence of clinical trial activities for participants A-E.

#### Study Equipment

**Figure 2:**
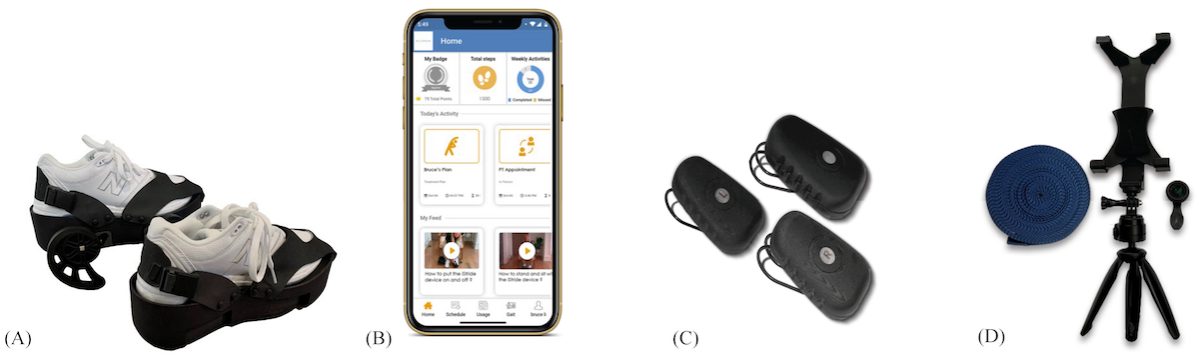
Study equipment: (A) iStride gait device and leveler (Moterum Technologies, Inc); (B) the Moterum Digital Platform; (C) Moterum sensors; (D) accessory equipment including a clip-on wide-angle camera lens, tablet tripod, and premeasured strap.

The following study equipment was provided to all participants: the iStride gait device, the leveler, access to the Moterum Digital Platform (the validated telerehabilitation platform [21-23] used in this study), the Moterum sensors (wearable gait sensors), and accessory treatment items. The gait device, shown in Figure 2, is worn on the foot of the nonparetic lower extremity during treatment. The device leveler, also shown in Figure 2, is a height-matched platform that is worn on the foot of the paretic lower extremity to equalize the added height of the gait device. The telerehabilitation platform (Figure 2) is a clinician-guided platform used to facilitate remote rehabilitation treatment. The platform is compliant with the Health Insurance Portability and Accountability Act (HIPAA). To support telerehabilitation, the platform incorporates live teleconferencing and can be used from any WiFi-enabled device (such as a tablet, smartphone, laptop, or computer). Treatment plans can be developed and programmed into the platform by a clinician to deliver personalized rehabilitation treatment. Participants were permitted to use their own device and were guided through instructions to download the rehabilitation platform before beginning the treatment. The gait sensors (Figure 2) are comprised of 3 inertial sensors (one worn on each shoe and a third worn on the waist) designed to track gait and movement patterns. Sensor data is transmitted to the rehabilitation platform, where it can be viewed by participants and clinicians. Participants also received accessory treatment equipment (Figure 2), including a clip-on wide-angle camera lens and tablet tripod to enhance the clinician’s view during sessions. Additionally, a premeasured strap with measured markings was provided to facilitate the accuracy of gait distance measurements during assessments.

#### Virtual Training

Participants and their care partners engaged in a remote training session provided by their physical therapist or a study team member using teleconferencing prior to beginning treatment. The session lasted approximately 60 minutes and included the following training topics: management of study equipment (including downloading and accessing the telerehabilitation app, donning and doffing the gait device and leveler and securing all straps, proper placement of sensors, and sensor charging instructions), comprehensive safety precautions, including (but not limited to) the need for care partner presence during all treatment activities, safe environmental conditions (clear walking path, adequate lighting, etc), guarding of the participants during treatment, activity pacing, indications for rests breaks, and using the Rating of Perceived Exertion (RPE) scale [24] to monitor perceived exertion during the treatment sessions. After the training session, individuals were provided with and required to pass a web-based quiz to verify their understanding of safety guidelines and expectations.

#### Assessment Sessions

Each participant underwent an assessment session to capture baseline mobility levels before beginning treatment. Assessments included the 10-Meter Walk Test (10MWT) at a comfortable walking speed, the Timed Up and Go Test (TUG), the 6-Minute Walk Test (6MWT), and the Stroke Specific Quality of Life Scale (SS-QOL). The 10MWT was used to measure comfortable walking speed. Functional mobility and risk for falls were assessed using the TUG Test. The 6MWT was used to measure aerobic endurance, and the SS-QOL was used to assess the participant's quality of life related to their stroke symptoms. These outcome measures were performed at baseline, after 1 month of treatment (time 1), approximately halfway through the treatment (time 2), and after the conclusion of treatment (post). All assessment sessions were conducted within each participant’s home environment, with their respective physical therapist providing virtual oversight using real-time video conferencing.

To enhance the accuracy of performing gait-focused outcome measures using telehealth, a 10-meter strap labeled with markings indicating the appropriate measurement distances for the 10MWT and the TUG Test was used. For the 6MWT, the test was adapted to the home environment using the 10-meter strap to calculate the distance (using the number of laps and partial laps) each participant could ambulate within 6 minutes. Note that these are not the standard testing procedures for this assessment [25], and studies show that performing the 6MWT on shorter courses (such as in home environments) may underestimate functional endurance [26,27]. Therefore, the participants’ aerobic capacity may be higher than stated. For each participant, the setup was consistent across all assessment sessions to enable accurate within-subjects comparison. Finally, the SS-QOL was provided in survey form, with responses summed to calculate a total score. Instructions for testing procedures were incorporated into the rehabilitation platform and therapist documentation forms for consistency.

#### Gait Monitoring

In addition to the gait device treatment and assessment sessions, participants were instructed to perform approximately 5 minutes of walking wearing the gait sensors. Data from these walks were used to further evaluate the participant’s ability to consistently use the sensor equipment. While changes to specific gait parameters were not included as an outcome in this study, gait data gathered by the sensor uploads were available in the telerehabilitation app for clinicians to monitor for potential gait pattern changes in response to treatment. Participants were provided with the goal of performing 5 minutes of walking with the sensors on 50% of days.

#### Treatment Sessions

Treatment occurred in each participant’s home and was facilitated by a physical therapist and the participant’s trained care partner. During the first treatment session, the physical therapist was virtually present using the teleconferencing feature incorporated within the rehabilitation platform. The therapists continued to virtually oversee the treatment sessions until the therapist, participant, and care partner all felt comfortable performing these sessions without therapist oversight. Four participants were provided a treatment plan of walking on the gait device 3 times per week for 30 minutes, and 1 was prescribed 5 times per week for 15 minutes. Participants were instructed to wear their gait sensors during all treatment and assessment sessions.

### Data Analysis: Feasibility Assessment

#### Overview

The feasibility assessment comprised evaluations of safety, compliance with protocol activities, user acceptability, and preliminary efficacy indicators. Participant safety, protocol compliance, and acceptability were defined as the primary outcomes. Feasibility success criteria specified no serious adverse events [28], average protocol activity compliance of >80% [29], and >60% positive acceptability responses. Secondary outcomes included indicators of efficacy, as determined by changes in the 10MWT, TUG, 6MWT, and SS-QOL assessments.

#### Safety and Treatment Tolerance

To determine the safety and tolerance of the remote treatment, participants and care partners were routinely questioned on the following: (1) the presence of falls; (2) whether they felt adequately supported by their care partner during treatment; (3) the highest level of assistance provided by their care partner during their treatment sessions; (4) the highest RPE during their treatment sessions; and (5) the level of pain before, during, and after treatment (using a numerical pain scale of 0-10, with higher numbers indicating increasing pain). Responses to these questions were aggregated and assessed for the range of responses and averages.

#### Compliance With Protocol Activities

To determine the participant’s willingness and ability to comply with the remote treatment, we evaluated the percentage of completion of the following tasks: (1) outcome measure assessment sessions, (2) treatment sessions, (3) sensor usage during treatment sessions, and (4) daily, 5-minute sensor walks. Tracking of these activities was maintained within the rehabilitation platform.

#### User Acceptability

After completing clinical trial activities, participants were emailed a questionnaire to assess their acceptability of the telehealth treatment. The questions included perceptions of treatment safety, acceptability of the telehealth delivery model, and observations of potential functional improvement. Acceptability was determined by the percentage of positive responses.

#### Efficacy Indicators

The participants’ gait was remotely monitored using functional outcome measures. Outcome measure changes were reviewed in comparison to minimal clinically important difference (MCID) or minimal detectable change (MDC) thresholds reported in the literature. MCID values are available in the population of stroke for the 10MWT (0.16 meters per second) [30] and the 6MWT (34.4 meters) [31]. An MDC threshold has been established in the population of patients with chronic stroke for the TUG at –3.2 seconds [32]. The SS-QOL does not have a total score MDC or MCID; therefore, the changes are reported without reference to such a threshold.

We additionally compared participants’ 10MWT results to gait speed classifications, which are used to reflect an individual’s ability to participate in mobility-related activities [33]. “Household ambulators” correspond to speeds <0.4 meters per second, “limited community ambulators” correspond to speeds between 0.4 meters per second and 0.8 meters per second, “unlimited community ambulators” are categorized with speeds >0.8 meters per second, and “normal speeds” are typically >1.2 meters per second [33,34]. Finally, a TUG Test threshold value of 13.5 seconds [35] is commonly used to separate high and low fall risk. To determine if the participants changed their fall risk classification during their gait treatment, TUG scores were compared to this threshold at pre- and posttreatment timeframes.

## Results

### Participants

Table 1 shows the demographic information of the 5 study participants. A sixth participant began the clinical trial but did not complete the study period; therefore, their information was not included in the analysis. During their period of participation, the sixth participant completed all scheduled assessments and treatment sessions, and no injuries occurred.

**Table 1:** Participant demographics.

ID	Age (years)	Sex	Weight (lbs)	Time since stroke (months)	Side of hemiparesis	Type of stroke	Device sessions before the clinical trial, n
A	60	Female	140	56	Left	Hemorrhagic	14
B	70	Male	160	132	Right	Ischemic	10
C	77	Female	170	46	Right	Ischemic	6
D	80	Male	230	48	Left	Ischemic	8
E	73	Female	147	138	Left	Hemorrhagic	0
Average	72.0	2 males, 3 females	169.4	84.0	3 left; 2 right	2 hemorrhagic; 3 ischemic	N/A^a^

^a^N/A: not applicable.

### Safety and Adverse Events

#### Safety Questionnaire and Adverse Events Report

One adverse event occurred out of 224 treatment sessions during the clinical trial. During this event, the participant’s pet became entangled in their legs, resulting in a slow, controlled fall. The participant and pet were uninjured. Additional education was provided to ensure pets were safely secured during treatment with the gait device. Responses to safety-related posttreatment questions and adverse events are available in Table 2.

**Table 2:** Safety and adverse events.

ID	Adverse events (falls)	Felt adequately supported by CG^a^ (% yes)	Highest level of CG assistance	Treatment sessions, n
A	1 (pet interference)	100	Contact guard assistance	39
B	0	100	Minimal assistance	74
C	0	100	Supervision/stand-by assistance	36
D	0	100	Contact guard assistance	40
E	0	100	Moderate assistance	35

^a^CG: caregiver.

#### Treatment Tolerance

Participants and their care partners recorded treatment tolerance information in accordance with their treatment sessions. Treatment tolerance information is available for review in Table 3.

**Table 3:** Treatment tolerance.

ID	Highest Rating of Perceived Exertion score, mean^a^	Increased pain (number of sessions)	Pain: before treatment, mean	Pain: during treatment, mean	Pain: after treatment, mean
A	3.0	0	0.0	0.0	0.0
B	4.0	0	0.0	0.0	0.0
C	4.1	0	0.0	0.0	0.0
D	4.2	0	3.7	3.7	3.7
E	5.2	0	1.1	1.1	0.9

^a^Modified Rating of Perceived Exertion scale (scoring 1-10).

### Compliance With Protocol Activities

As described above, the telehealth treatment delivery protocol included mobility assessments, treatment sessions, 5-minute walks, and requests to wear the gait sensors during the described mobility activities. Participants were evaluated on their percentage of compliance with these requested activities, as seen in Table 4. Note that compliance for the sensor walks was based on a target of 50% of days. Participants B and D performed this activity at a greater frequency; therefore, their compliance on this activity, and participant B’s average compliance across all activities, exceeds 100%. The headings *month 1*, *month 2*, and *final* in Table 4 report the compliance for the first, second, and final months of treatment, respectively.

**Table 4:** Compliance with protocol activities.

ID	Treatment sessions (%)			Monthly outcomes (%)			Sensors during treatment (%)			Sensor walks (%)	Mean (%)
	Month 1	Month 2	Final	Month 1	Month 2	Final	Month 1	Month 2	Final	Peer week^a^	
A	100	100	100	100	100	100	88	100	94	94.3	97.6
B	90	100	100	100	100	100	100	100	94	157.1	104.1
C	100	100	91	100	100	100	66	92	91	57.1	87.7
D	86	100	93	100	100	100	66.7	60	44	0	75.0
E	100	73	92	100	100	100	100	91	92	105.7	95.4
Mean (%)	95.2	94.6	95.2	100	100	100	84.1	88.6	83.0	82.8	N/A^b^

^a^Percentage is based on target of 50% of days.

^b^N/A: not applicable.

### Acceptability

Table 5 presents responses to a questionnaire designed to assess the acceptability of telehealth treatment delivery. Available answer choices are provided along with percentages of participants that selected each response.

**Table 5:** Acceptability questionnaire responses and percentages of selected responses.

Questions	Responses
The amount of effort required from my caregiver and I was…	“Reasonable” (100%) “Too much” (0%) “I’m not sure” (0%)
During my treatment sessions, I felt…	“Safe. My caregiver was able to provide adequate support while I walked on the [gait device].” (100%) “Unsafe. I needed more support than my caregiver could provide.” (0%) “Neither safe nor unsafe.” (0%)
After using the gait device, my walking feels…	“Somewhat improved” (60%) “Significantly improved” (40%) “Not improved” (0%) “Worsened” (0%) “I’m not sure” (0%)
I would recommend treatment with the gait device to others in a similar situation as myself…	“Strongly agree” (60%) “Agree” (40%) “Neither agree nor disagree” (0%) “Disagree” (0%) “Strongly disagree” (0%)
If given the opportunity, would you want to continue using the gait device with telehealth delivery? (As you have done during this clinical trial)?	“Yes” (100%) “No” (0%)

### Preliminary Efficacy

#### Outcome Measure Results

**Figure 3:**
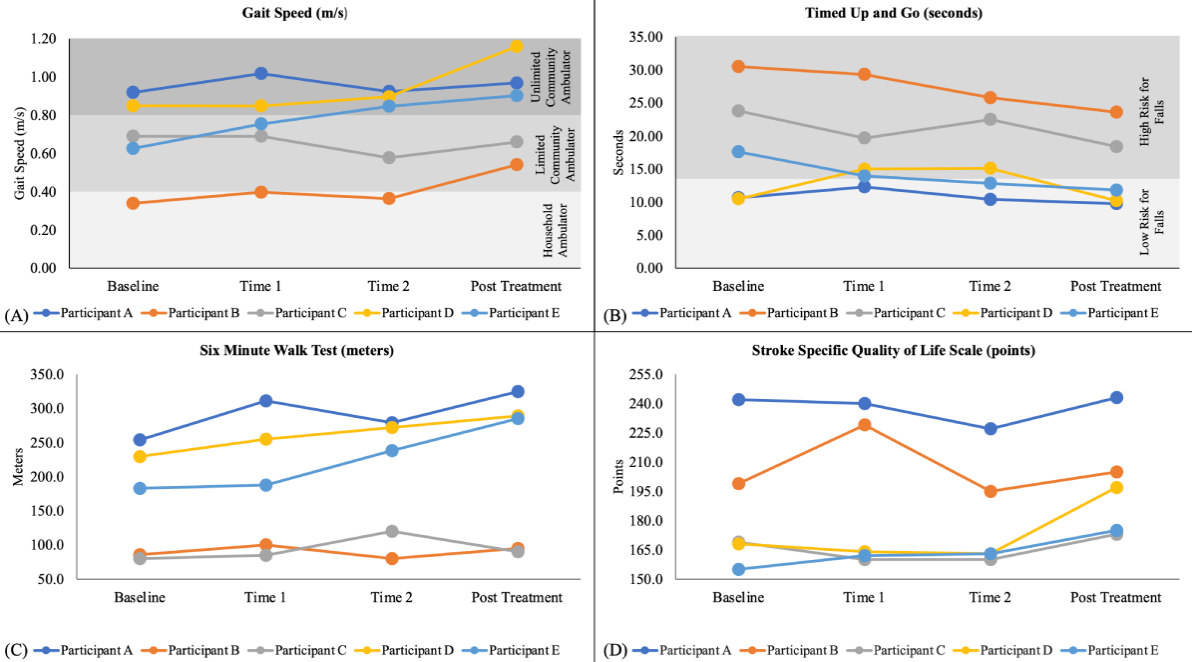
(A) Gait speed throughout the remote treatment period. Background shading represents different gait speed classification categories [33]. (B) Time Up and Go (TUG) scores throughout the remote treatment period. Background shading indicates the corresponding fall risk status [35]. (C) 6-Minute Walk Test (6MWT) scores throughout the remote treatment period. (D) Stroke Specific Quality of Life Scale (SS-QOL) scores throughout the remote treatment period.

Figure 3 shows the participants’ results on the outcome measurements of gait speed, the TUG Test, the 6MWT, and the SS-QOL Scale at each of the study periods.

#### Individual Comparison to Improvement Thresholds (MCID and MDC)

Table 6 compares outcome measure changes to each outcome’s MCID or MDC threshold of improvement (referenced within the table) after approximately 3 months of remote treatment with the gait device.

**Table 6:** Efficacy of treatment and comparison to MCID^a^ and MDC^b^ thresholds.

ID	10MWT^c^ (m/s) (MCID=0.16 m/s [30])	TUG^d^ (s) (MDC=–3.2 s [32])	6MWT^e^ (m) (MCID=34.4 m [31])	SS-QOL^f^
A	0.05	–0.90	*70.87^g^*	1.0
B	*0.20^g^*	*–6.90^g^*	9.50	6.0
C	–0.03	*–5.40^g^*	10.00	4.0
D	*0.31^g^*	–0.28	*59.50^g^*	29.0
E	*0.28^g^*	*–5.78^g^*	*102.00^g^*	20.0
Average	*0.16^g^*	*–3.85^g^*	*50.37^g^*	12.0

^a^MCID: minimal clinically important difference.

^b^MDC: minimal detectable change.

^c^10MWT: 10-Meter Walk Test.

^d^TUG: Timed Up and Go.

^d^MDC: minimal detectable change.

^e^6MWT: 6-Minute Walk Test.

^f^SSQOL: Stroke Specific Quality of Life Scale. No total score MCID or MDC has been identified in the literature.

^g^Indicates improvement beyond the MCID or MDC value.

## Discussion

### Principal Findings

This study investigates the feasibility of 2 telehealth delivery models for remote treatment with a home-use gait device, adapted in response to the onset of the COVID-19 pandemic. In the first method (participants A-D), existing gait device users transitioned into a remote telehealth delivery model to continue their gait treatment remotely in accordance with COVID-19 pandemic guidelines. In the second method (participant E), all processes, from recruitment through treatment, were delivered remotely. The results demonstrate promising indicators of feasibility, including safety, tolerance, compliance, and acceptability, as well as a suggestion of efficacy, for both remote delivery models. Moreover, these results inform various remote treatment methodologies that may be further explored or refined in future contexts, expanding treatment opportunities for those requiring or preferring remote treatment or enabling more frequent treatment for those with appropriately trained care partner support.

From a safety standpoint, no serious adverse events or injuries occurred throughout the clinical trial, and the protocol was well-tolerated by the participants. These findings occurred despite all therapists interacting remotely throughout the clinical trial period. Compliance with the assessed activities also remained high throughout the 3-month study period. On an individual basis, 4 out of the 5 participants exceeded 85% average compliance across all activities, and the remaining participant achieved 75% compliance. Regarding specific activities, activities using gait sensors demonstrated the weakest compliance but remained above 80% on average. This finding suggests that methods to enhance compliance, such as personal reminders, push notifications, or other engineering adaptations within the telerehabilitation platform, could be further investigated.

Responses to an acceptability questionnaire at the conclusion of treatment indicate a high level of participant acceptability for the telehealth methodologies. Specifically, all participants, including those that had previously experienced in-person treatment with the gait device, rated that they would want to use the gait device again using telehealth if given the opportunity. Additionally, all “agreed” or “strongly agreed” that they would recommend this treatment to others in a similar situation as themselves. Importantly, all participants felt that their walking was either somewhat or substantially improved, highlighting a fundamental indication of perceived efficacy. Given the lack of guidance from published studies regarding using telehealth for gait assessment and treatment, these highly favorable responses demonstrate that these methodologies are of value to the participants and worthy of continued exploration and development.

All participants improved by a clinically meaningful amount on at least 1 functional outcome, and 3 out of 5 participants improved clinically in each category at the final timeframe. Additionally, the average improvement on 3 out of 4 efficacy indicators surpassed MDC or MCID values, indicating a change that exceeded measurement error or was clinically meaningful to the individual [30-32]. These functional improvement results are particularly of interest as 4 out of the 5 participants previously completed between 6 and 14 in-person gait device treatment sessions before starting this clinical trial. Their continued improvement suggests that a more extended treatment duration, facilitated by transitioning to treatment with a care partner and supervision via telehealth, as depicted in this study, may be beneficial in addition to the shorter protocol described in our prior studies [15,18].

Participant E, the only participant who received no prior gait device treatment before the clinical trial, attained considerable improvements across all functional outcome measures. These results include a 0.28 meters per second gait speed improvement that corresponds with an improved gait speed classification from limited to unlimited community ambulator [33]. This change reflects expected improved community participation ability, an important attribute associated with enhanced quality of life in individuals with stroke [36] (further emphasized by the 20-point SS-QOL improvement). Participant E also attained a 5.78-second improvement on the TUG Test, consistent with a reduction in fall risk from high to low [35], and a >100-meter improvement on the 6MWT. These functional gains substantially exceed the respective MCID or MDC thresholds. Additionally, these improvements exceed the average improvements from our previous 4-week in-person gait device study [15], likely reflecting the benefits of the increased treatment duration. While the majority of patients are able to begin treatment with in-home visits from a physical therapist, the positive results of this participant suggest that fully remote treatment with the gait device can be safe and efficacious for some individuals with chronic stroke and care partner support.

Improvement of the SS-QOL, which does not have an accepted total score MDC, was moderate (12 points; total possible scores for this outcome range from 49-245) compared to the other outcomes. However, with the study period encompassing the summer and fall months of 2020, it seems plausible that the events and consequences of the COVID-19 pandemic may have influenced these findings. As researchers continue to investigate the effects of the COVID-19 pandemic on various aspects of health and well-being, studies have begun to highlight the particularly devastating effects of the COVID-19 pandemic on individuals with disabilities [37,38]. It is possible that disproportionate pandemic consequences may have limited improvement in SS-QOL scores for some participants in our study. Nevertheless, the absence of severe adverse events, high compliance across all protocol activities, positive acceptability responses, and a preliminary suggestion of efficacy support the feasibility of the remote telehealth treatment protocols.

### Limitations

Several limitations should be acknowledged in this feasibility study. First, the small sample size is a limitation. While the study does include a wide range of baseline ambulatory abilities (baseline gait speed from approximately 0.3 meters per second to 0.9 meters per second, encompassing gait classifications of household ambulator, limited community ambulator, and unlimited community ambulator), the small sample size limits both generalizability and confirmation of efficacy. Additionally, the unmatched group sizes between the 2 telehealth treatment methodologies prevent statistical comparisons between the groups. In addition, our population was restricted to individuals with consistent care partner support and those whom the consulting therapists believed could perform the treatment safely. These restrictions limit the population of individuals to which this study may provide relevance. Additionally, all study participants were individuals who sought out (or previously participated in) treatment with the gait device, which may have presented a more compliant sample than the general population.

While there are many benefits to providing in-home treatment, ambulation assessment is challenging in comparison to clinical environments. The 6MWT was included in this study based on subjective comments from prior gait device users that reported improved functional endurance post treatment. In agreement with these reports, the average 6MWT improvement exceeded 50 meters, with 1 participant exceeding 100 meters of improvement. However, it is important to note that the 6MWT was adapted to the home environment from its recommended protocol [25]. Therefore, the interpretation of the 6MWT findings and a direct comparison to a clinically obtained MCID threshold warrants caution.

The amount of therapist interaction or “virtual” presence during treatment sessions, which was based on the individual therapist’s clinical judgment, was not controlled, as it was of more importance to let the therapist assess each individual’s safety. Additionally, we did not require that participants abstain from external therapy services during the study period (although, to our knowledge, only 1 participant received brief services to address dizziness).

### Conclusions

In conclusion, the results of this study demonstrate the feasibility of remote treatment with the gait device. Participants A-D attained continued functional improvement after transitioning to telehealth, indicating a possible benefit for both extended treatment duration and combining in-person and remote treatment methodologies. Participant E, who performed all activities remotely, improved clinically on all outcomes, indicating that fully remote treatment may be a suitable option for some individuals needing or desiring virtual care. Larger, future studies could further confirm these findings as well as clarify characteristics for a broader population that may benefit from this treatment delivery. At present, these promising results indicate that telehealth can be a feasible delivery method for treatment with the gait device. Moreover, the formative nature of this work has provided support for further exploration and insight to refine remote and hybrid methodologies, which may serve to expand treatment opportunities for those experiencing gait impairments attributed to neurologic conditions.

## Appendix

Multimedia Appendix 1 Outcome measures data set.

## Abbreviations

10MWT: 10-Meter Walk Test
6MWT: 6-Minute Walk Test
CDC: Centers for Disease Control and Prevention
CONSORT: Consolidated Standards of Reporting Trials
HIPAA: Health Insurance Portability and Accountability Act
MCID: minimal clinically important difference
MDC: minimal detectable change
RPE: Rating of Perceived Exertion
SS-QOL: Stroke Specific Quality of Life
TUG: Timed Up and Go

## Data Availability

The data generated or analyzed during this study are included in this paper and in Multimedia Appendix 1.

## References

[1] Impact of COVID-19 on the physical therapy profession. American Physical Therapy Association.

[2] Doraiswamy S, Abraham A, Mamtani R, Cheema S (2020). Use of telehealth during the COVID-19 pandemic: scoping review. J Med Internet Res.

[3] Ku BPS, Tse AWS, Pang BCH, Cheung NT, Pang JYW, Chan JKY, Hui HL, Chu D, Choi KHW (2021). Tele-rehabilitation to combat rehabilitation service disruption during COVID-19 in Hong Kong: observational study. JMIR Rehabil Assist Technol.

[4] Rehabilitation considerations during the COVID-19 outbreak. Pan American Health Organization.

[5] Chang MC, Boudier-Revéret M (2020). Usefulness of telerehabilitation for stroke patients during the COVID-19 pandemic. Am J Phys Med Rehabil.

[6] Aggarwal G, Lippi G, Michael Henry B (2020). Cerebrovascular disease is associated with an increased disease severity in patients with coronavirus disease 2019 (COVID-19): a pooled analysis of published literature. Int J Stroke.

[7] Appleby E, Gill ST, Hayes LK, Walker TL, Walsh M, Kumar S (2019). Effectiveness of telerehabilitation in the management of adults with stroke: a systematic review. PLoS One.

[8] Cramer SC, Dodakian L, Le V, See J, Augsburger R, McKenzie A, Zhou RJ, Chiu NL, Heckhausen J, Cassidy JM, Scacchi W, Smith MT, Barrett AM, Knutson J, Edwards D, Putrino D, Agrawal K, Ngo K, Roth EJ, Tirschwell DL, Woodbury ML, Zafonte R, Zhao W, Spilker J, Wolf SL, Broderick JP, Janis S, National Institutes of Health StrokeNet Telerehab Investigators (2019). Efficacy of home-based telerehabilitation vs in-clinic therapy for adults after stroke: a randomized clinical trial. JAMA Neurol.

[9] Cacciante L, Kiper P, Garzon M, Baldan F, Federico S, Turolla A, Agostini M (2021). Telerehabilitation for people with aphasia: a systematic review and meta-analysis. J Commun Disord.

[10] Tchero H, Tabue Teguo M, Lannuzel A, Rusch E (2018). Telerehabilitation for stroke survivors: systematic review and meta-analysis. J Med Internet Res.

[11] Guo CC, Chiesa PA, de Moor C, Fazeli MS, Schofield T, Hofer K, Belachew S, Scotland A (2022). Digital devices for assessing motor functions in mobility-impaired and healthy populations: systematic literature review. J Med Internet Res.

[12] Li S, Francisco GE, Zhou P (2018). Post-stroke hemiplegic gait: new perspective and insights. Front Physiol.

[13] Dobkin BH (2005). Clinical practice. Rehabilitation after stroke. N Engl J Med.

[14] Reed KB, Handzic I Gait altering shoes. University of South Florida, assignee.

[15] Huizenga D, Rashford L, Darcy B, Lundin E, Medas R, Shultz ST, DuBose E, Reed KB (2020). Wearable gait device for stroke gait rehabilitation at home. Top Stroke Rehabil.

[16] Handzic I, Vasudevan E, Reed KB (2012). Developing a gait enhancing mobile shoe to alter over-ground walking coordination. IEEE Int Conf Robot Autom.

[17] Handzic I, Barno EM, Vasudevan EV, Reed KB (2011). Design and pilot study of a gait enhancing mobile shoe. Paladyn.

[18] Kim SH, Huizenga DE, Handzic I, Ditwiler RE, Lazinski M, Ramakrishnan T, Bozeman A, Rose DZ, Reed KB (2019). Relearning functional and symmetric walking after stroke using a wearable device: a feasibility study. J Neuroeng Rehabil.

[19] (2020). Public health activity guidance. Center for Disease Control and Prevention.

[20] Eldridge SM, Chan CL, Campbell MJ, Bond CM, Hopewell S, Thabane L, Lancaster GA, PAFS consensus group (2016). CONSORT 2010 statement: extension to randomised pilot and feasibility trials. Pilot Feasibility Stud.

[21] Lee K, Nathwani N, Shamunee J, Lindenfeld L, Wong FL, Krishnan A, Armenian S (2022). Telehealth exercise to Improve Physical function and frailty in patients with multiple myeloma treated with autologous hematopoietic Stem cell transplantation (TIPS): protocol of a randomized controlled trial. Trials.

[22] Landers MR, Ellis TD (2020). A mobile app specifically designed to facilitate exercise in Parkinson disease: single-cohort pilot study on feasibility, safety, and signal of efficacy. JMIR Mhealth Uhealth.

[23] Sarfo FS, Adusei N, Ampofo M, Kpeme FK, Ovbiagele B (2018). Pilot trial of a tele-rehab intervention to improve outcomes after stroke in Ghana: a feasibility and user satisfaction study. J Neurol Sci.

[24] Borg G (1970). Perceived exertion as an indicator of somatic stress. Scand J Rehabil Med.

[25] ATS Committee on Proficiency Standards for Clinical Pulmonary Function Laboratories (2002). ATS statement: guidelines for the six-minute walk test. Am J Respir Crit Care Med.

[26] Ng SS, Tsang WW, Cheung TH, Chung JS, To FP, Yu PC (2011). Walkway length, but not turning direction, determines the six-minute walk test distance in individuals with stroke. Arch Phys Med Rehabil.

[27] Beekman E, Mesters I, Hendriks EJM, Klaassen MPM, Gosselink R, van Schayck OCP, de Bie RA (2013). Course length of 30 metres versus 10 metres has a significant influence on six-minute walk distance in patients with COPD: an experimental crossover study. J Physiother.

[28] What is a serious adverse event?. Food and Drug Administration.

[29] Moore CG, Carter RE, Nietert PJ, Stewart PW (2011). Recommendations for planning pilot studies in clinical and translational research. Clin Transl Sci.

[30] Tilson JK, Sullivan KJ, Cen SY, Rose DK, Koradia CH, Azen SP, Duncan PW, Locomotor Experience Applied Post Stroke (LEAPS) Investigative Team (2010). Meaningful gait speed improvement during the first 60 days poststroke: minimal clinically important difference. Phys Ther.

[31] Tang A, Eng JJ, Rand D (2012). Relationship between perceived and measured changes in walking after stroke. J Neurol Phys Ther.

[32] Alghadir AH, Al-Eisa ES, Anwer S, Sarkar B (2018). Reliability, validity, and responsiveness of three scales for measuring balance in patients with chronic stroke. BMC Neurol.

[33] Perry J, Garrett M, Gronley JK, Mulroy SJ (1995). Classification of walking handicap in the stroke population. Stroke.

[34] Fritz S, Lusardi M (2009). White paper: "Walking speed: the sixth vital sign". J Geriatr Phys Ther.

[35] Shumway-Cook A, Brauer S, Woollacott M (2000). Predicting the probability for falls in community-dwelling older adults using the Timed Up & Go test. Phys Ther.

[36] Khanittanuphong P, Tipchatyotin S (2017). Correlation of the gait speed with the quality of life and the quality of life classified according to speed-based community ambulation in Thai stroke survivors. NeuroRehabilitation.

[37] Lebrasseur A, Fortin-Bédard N, Lettre J, Bussières EL, Best K, Boucher N, Hotton M, Beaulieu-Bonneau S, Mercier C, Lamontagne M, Routhier F (2021). Impact of COVID-19 on people with physical disabilities: a rapid review. Disabil Health J.

[38] Constantino JN, Sahin M, Piven J, Rodgers R, Tschida J (2020). The impact of COVID-19 on individuals with intellectual and developmental disabilities: clinical and scientific priorities. Am J Psychiatry.

