# Supplementary material for: Gait Device Treatment Using Telehealth for Individuals With Stroke During the COVID-19 Pandemic: Nonrandomized Pilot Feasibility Study
Source: JMIR Form Res. 2023 May 19;7:e43008. doi: 10.2196/43008 (PMC10238959; doi:10.2196/43008)
Supplement: Multimedia Appendix 1 [file formative_v7i1e43008_app1.docx]

Multimedia Appendix 1. Outcome measures data set.

| **Gait Speed (meters/second)** | | | | | | | | | | | | | |  |  |  |
| --- | --- | --- | --- | --- | --- | --- | --- | --- | --- | --- | --- | --- | --- | --- | --- | --- |
| **ID** | **Baseline** | | **Time 1** | | | **Time 2** | | | | **Post-Treatment** | | | |  |  |  |
| A | 0.92 | | 1.02 | | | 0.92 | | | | 0.97 | | | |  |  |  |
| B | 0.34 | | 0.40 | | | 0.36 | | | | 0.54 | | | |  |  |  |
| C | 0.69 | | 0.69 | | | 0.58 | | | | 0.66 | | | |  |  |  |
| D | 0.85 | | 0.85 | | | 0.90 | | | | 1.16 | | | |  |  |  |
| E | 0.63 | | 0.75 | | | 0.85 | | | | 0.90 | | | |  |  |  |
|  |  | | | |  | | |  | | |  |  |  |  |  |  |
| **Timed Up and Go Test (seconds)** | | | | | | | | | | | | | |  |  |  |
| **ID** | **Baseline** | | **Time 1** | | | **Time 2** | | | | **Post-Treatment** | | | |  |  |  |
| A | 10.7 | | 12.3 | | | 10.4 | | | | 9.8 | | | |  |  |  |
| B | 30.5 | | 29.3 | | | 25.8 | | | | 23.6 | | | |  |  |  |
| C | 23.8 | | 19.7 | | | 22.5 | | | | 18.4 | | | |  |  |  |
| D | 10.5 | | 15.0 | | | 15.1 | | | | 10.2 | | | |  |  |  |
| E | 17.6 | | 13.9 | | | 12.8 | | | | 11.8 | | | |  |  |  |
|  |  | | | |  | | |  | | |  |  |  |  |  |  |
| **Six Minute Walk Test (meters)** | | | | | | | | | | | | | |  |  |  |
| **ID** | **Baseline** | | **Time 1** | | | **Time 2** | | | | **Post-Treatment** | | | |  |  |  |
| A | 253.7 | | 310.9 | | | 278.9 | | | | 324.6 | | | |  |  |  |
| B | 85.5 | | 100.0 | | | 80.0 | | | | 95.0 | | | |  |  |  |
| C | 80.0 | | 85.0 | | | 120.0 | | | | 90.0 | | | |  |  |  |
| D | 229.5 | | 255.0 | | | 272.0 | | | | 289.0 | | | |  |  |  |
| E | 183.0 | | 187.5 | | | 238.4 | | | | 285.0 | | | |  |  |  |
|  |  | | | |  | | |  | | |  |  |  |  |  |  |
| **Stroke Specific Quality of Life Scale (points)** | | | | | | | | | | | | | |  |  |  |
| **ID** | **Baseline** | | **Time 1** | | | **Time 2** | | | | **Post-Treatment** | | | |  |  |  |
| A | 242 | | 240 | | | 227 | | | | 243 | | | |  |  |  |
| B | 199 | | 229 | | | 195 | | | | 199 | | | |  |  |  |
| C | 169 | | 160 | | | 160 | | | | 173 | | | |  |  |  |
| D | 168 | | 164 | | | 163 | | | | 197 | | | |  |  |  |
| E | 155 | | 162 | | | 163 | | | | 175 | | | |  |  |  |
|  | |  | |  | | |  | |  | | | |  | |  |  |
